# Sources of variability in seagrass fatty acid profiles and the need of identifying reliable warming descriptors

**DOI:** 10.1038/s41598-023-36498-2

**Published:** 2023-06-20

**Authors:** Arianna Pansini, Pedro Beca-Carretero, Maria J. González, Gabriella La Manna, Isabel Medina, Giulia Ceccherelli

**Affiliations:** 1 Dipartimento di Scienze Chimiche Fisiche Matematiche e Naturali, Università Degli Studi di Sassari, Via Piandanna 4, 07100 Sassari, Italy; 2 Department of Oceanography, Instituto de Investigacións Mariñas (IIM-CSIC), 36208 Vigo, Spain; 3 MareTerra Onlus, Environmental Research and Conservation, 07041 Alghero, SS Italy

**Keywords:** Fatty acids, Environmental impact, Marine biology

## Abstract

Global warming is expected to have inexorable and profound effects on marine ecosystems, particularly in foundation species such as seagrasses. Identifying responses to warming and comparing populations across natural temperature gradients can inform how future warming will impact the structure and function of ecosystems. Here, we investigated how thermal environment, intra-shoot and spatial variability modulate biochemical responses of the Mediterranean seagrass *Posidonia oceanica*. Through a space-for-time substitution study, Fatty acid (FA) profiles on the second and fifth leaf of the shoots were quantified at eight sites in Sardinia along a natural sea surface temperature (SST) summer gradient (about 4 °C). Higher mean SST were related to a decrease in the leaf total fatty acid content (LTFA), a reduction in polyunsaturated fatty acids (PUFA), omega-3/omega-6 PUFA and PUFA/saturated fatty acids (SFA) ratios and an increase in SFA, monounsaturated fatty acids and carbon elongation index (CEI, C18:2 n-6/C16:2 n-6) ratio. Results also revealed that FA profiles were strongly influenced by leaf age, independently of SST and spatial variability within sites. Overall, this study evidenced that the sensitive response of *P. oceanica* FA profiles to intra-shoot and spatial variability must not be overlooked when considering their response to temperature changes.

## Introduction

Predicting the consequences of global warming and identifying mitigation and restoration actions in the future is critical, particularly in the sea^1–5^. Evaluating early warning stressor-specific responses of foundation species is pivotal to understand the effects of future warming and to predict their status, and consequently their structure and distribution. In this context, for marine macrophytes temperature is one of the most important limiting factors^6,7^.

Seagrasses, one of the most important and productive marine coastal ecosystems, have a key role in ecosystem functioning and services, providing blue carbon sequestration and nutrient cycling, forming habitats, nursery grounds, sediment stabilization, trophic transfer to other habitats^8–12^ and coastal protection from erosion^13,14^. Several local anthropogenic stressors, such as sediments and nutrient inputs, physical disturbance such as trawling, anchoring, invasions of exotic species have put seagrasses under high pressure over the past decades^15–19^. Moreover, concern is growing over the impacts of warming on seagrasses, increasing seagrass shoot mortality^20–22^ and limiting meadow recovery and structure^23^. Therefore, understanding their response to climate change is pivotal to determine their future ecosystem functioning and services. Thermal environment is likely to shape the structure and density of a meadow, driving morphological adaptations and acclimations^24–26^, physiology^6^, and reproductive phenology^27,28^. However, defining the effects of temperature changes on seagrass species remains a controversial topic that has not found unequivocal descriptions likely because results can be affected by local species adaptations making the acclimation responses difficult to measure and interpret^29–31^. However, depending on the intensity and duration of the heat, sublethal effects can be observed at different plant organization levels: changes in morphology may include leaf fall and leaf necrosis^32,33^, remodelling leaf biochemistry as carbohydrates and lipids^33–35^, and adjusting the transcriptomic regulation at molecular level^36,37^.

Several seagrass traits are widely used to assess coastal ecosystem status in the past years^6^, and among these the composition of Fatty acids (FA) can represent a valuable physiological descriptor. FA are vital structural compounds of primary producers, constitute the structure of the membranes of the thylakoids^38–40^ and play a fundamental role in the photosynthetic activity^41^. The remodelling of leaf FA due to temperature increase has been found in a wide range of plant species living in several biomes^42^ and in particular in seagrass species^40^. Results consistently indicate that heat reduces polyunsaturated fatty acids (PUFA), counterbalanced by the increase in saturated fatty acids (SFA) and monounsaturated fatty acids (MUFA, e.g.,^40,43,44^). However, although the use of FA analysis for understanding marine ecosystem functioning is promising, the variability in FA composition, either as single or grouped fatty acids, needs to be estimated to ensure relevant and appropriate interpretations^45^.

With the aim of evaluating the potential sources of variability in seagrass leaf FA composition at different environmental conditions, we realized that only relatively few studies have investigated variations in FA composition along natural environmental gradients^34,46,47^, while the majority have been conducted in mesocosms with artificial induced changes. Remodelling in FA profiles related to a heat stress, such as a reduction in PUFA, in total Fatty acids, and an increase in SFA^34,47^, is connected to the healthy functioning of photosynthetic activity, and consequently, to the plant resilience to the environmental changes.

Furthermore, the material collected for analyses is often inconsistent for seagrasses, even within samples belonging to the same species: most authors referred to generic leaf biomass of the same shoot both in *Posidonia oceanica* and *Zostera noltii* (e.g.^47,48^*.* and^49,50^ respectively), while recently only young leaves were considered (e.g.^39,43^*.* with *Zostera marina* apical shoots). Very little attention is paid to the specific leaf analysed: only Park et al.^51^ measured differences between fresh and senescent leaves of *Z. marina*, and Viso et al.^52^ compared chlorophyllous to non-chlorophyllous part of the same leaf in *P. oceanica*. However, because in each bundle there are differences across leaves, younger leaves internally enclosed by the external older leaves^9^, each leaf corresponds to a different time of production, exposition, morphology (leaf length, necrotic portions, broken apex^53^), and epiphytic cover^54^. Analysing the entire shoot material or focusing only on a specific leaf of the shoot could hinder some leaf-type functioning, metabolism, and phenology features. Previous evidence highlighted differences in biochemical composition and physiological processes along within-shoot leaf variability as in isotopic composition^55^, nutrient mass balance^56,57^, phenolic compounds^58,59^ and investigating intra-plant variation in FA composition will be crucial in determining the interaction outcomes between seagrasses and their surrounding environment. Thus, here we hypothesize that the FA composition would depend on the leaf age within the same shoot.

In this context, this study investigated the dependence of FA composition on a thermal gradient in the seagrass *Posidonia oceanica* using a space-for-time substitution approach, where the relationships between ecological variables are studied at sites assumed to be at different stages of development^60^. The study also estimated the variability in FA composition due to leaf material and spatial variability at a local scale, across the gradient. The first hypothesis is that FA profile varies along a temperature gradient resembling the responses to changes due to future warming, mainly with an expected decrease of PUFAs relative to SFAs at higher temperatures. A further approach was used to test the second hypothesis that FA profiles can depend also on intra-shoot variability by comparing the second vs fifth leaf of the same shoot, with an expected higher level of PUFAs in the former due to higher productivity. Lastly, we tested the third hypothesis that spatial variability among meadows reflects differences in local conditions rather than temperature. With these aims, we considered meadows along a natural gradient of water temperature in Sardinia (western Mediterranean Sea) that allowed examination of the association of *P. oceanica* FA composition for a wide range of Sea Surface Temperature (SST, about 4 °C) comparable to the increase in SST according to future warming scenarios^61^, with minimum interference of factors associated to a narrow latitudinal range (i.e., photoperiod). Overall, results provide knowledge about the substantial source of variability of seagrass leaf FA composition contributing to produce accurate sampling practices and monitoring procedures and rely on valuable warming descriptors.

## Results

**Figure 1:**
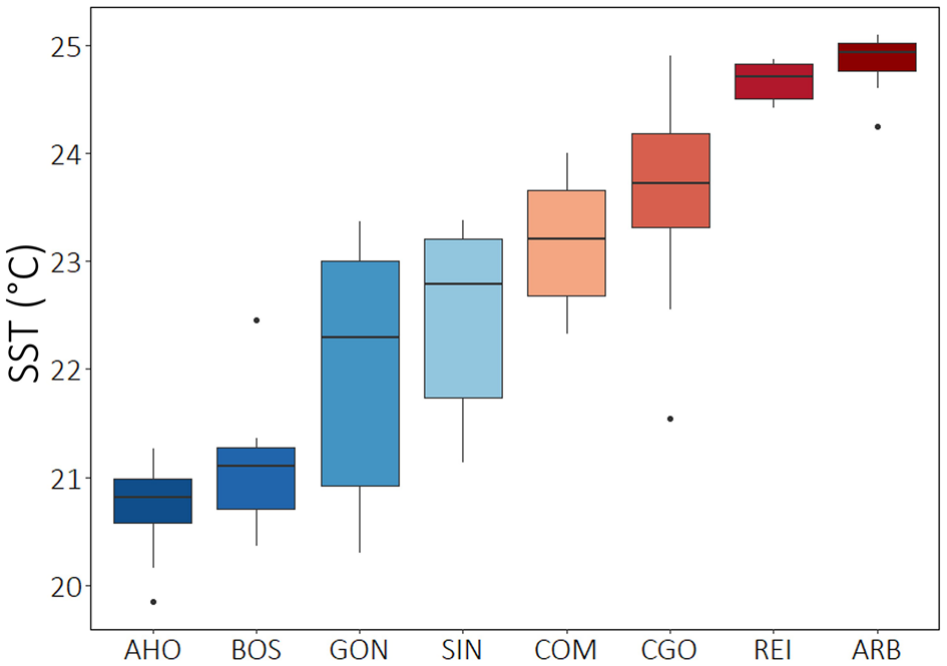
Boxplots of the Sea Surface Temperature (SST) daily measures over the ten days preceding the *Posidonia oceanica* shoot collection for each site.

The thermal environment at the eight sites in the 10 days before sampling was widely different, mean SST ranging from 20.72 °C (SE ±0.14) in AHO to 24.84 °C (SE ±0.26) in ARB (Table 1 and Fig. 1). On both Sardinia coastlines the northern sites were colder, but for the differences in temperature between coasts all sites were homogeneously placed within a gradient in temperature. The average values of FAs, both as single and grouped fatty acids, varied across sites and areas (Table 1 and Supplementary Fig. S2).

**Table 1:** Fatty acid composition (% of the total Fatty acids) and content (% of the leaf mg DW) expressed in mean ± SE at the sites: AHO = Alghero, BOS = Bosa, SIN = Penisola del Sinis, GON = Gonnesa, COM = Capo Comino, CGO = Cala Gonone, ARB = Arbatax, REI = Costa Rei.

Site	AHO	BOS	GON	SIN	COM	CGO	REI	ARB
SST (°C)	20.72	21.09	22.02	22.45	23.20	23.57	24.67	24.84
Saturated fatty acids								
C14:0	0.44 ± 0.06	0.51 ± 0.07	0.47 ± 0.03	0.54 ± 0.10	0.59 ± 0.04	0.61 ± 0.07	0.69 ± 0.05	0.98 ± 0.21
C15:0	0.16 ± 0.01	0.26 ± 0.03	0.19 ± 0.01	0.18 ± 0.02	0.23 ± 0.01	0.27 ± 0.02	0.21 ± 0.02	0.29 ± 0.02
C16:0	19.56 ± 0.36	24.00 ± 0.65	20.86 ± 0.61	20.29 ± 0.72	22.25 ± 0.39	23.11 ± 0.63	21.23 ± 0.43	25.76 ± 0.77
C18:0	2.64 ± 0.08	3.36 ± 0.12	3.12 ± 0.11	3.08 ± 0.12	3.73 ± 0.17	3.66 ± 0.17	3.19 ± 0.16	3.89 ± 0.18
C20:0	0.42 ± 0.03	0.53 ± 0.05	0.48 ± 0.03	0.41 ± 0.03	0.51 ± 0.01	0.53 ± 0.03	0.47 ± 0.02	0.58 ± 0.03
Sum of SFA	23.23 ± 0.46	28.67 ± 0.81	25.13 ± 0.72	24.51 ± 0.83	27.32 ± 0.47	28.18 ± 0.74	25.80 ± 0.57	31.51 ± 1.12
Monounsaturated fatty acids								
C16:1 n-9	0.19 ± 0.01	0.24 ± 0.02	0.30 ± 0.05	0.24 ± 0.03	0.30 ± 0.05	0.40 ± 0.04	0.29 ± 0.03	0.34 ± 0.03
C16:1 n-7	1.40 ± 0.09	1.27 ± 0.10	1.46 ± 0.07	1.19 ± 0.10	1.36 ± 0.11	1.53 ± 0.13	1.22 ± 0.07	1.57 ± 0.14
C18:1 n-9	2.08 ± 0.14	2.03 ± 0.19	2.52 ± 0.17	3.10 ± 0.35	2.86 ± 0.31	3.45 ± 0.37	2.60 ± 0.17	3.06 ± 0.27
C18:1 n-7	0.22 ± 0.02	0.28 ± 0.02	0.27 ± 0.02	0.26 ± 0.02	0.24 ± 0.01	0.27 ± 0.01	0.28 ± 0.01	0.31 ± 0.03
Sum of MUFA	3.89 ± 0.12	3.82 ± 0.18	4.55 ± 0.16	4.78 ± 0.33	4.76 ± 0.27	5.65 ± 0.33	4.39 ± 0.16	5.28 ± 0.35
Polyunsaturated fatty acids								
C16:2 n-6	0.41 ± 0.03	0.61 ± 0.09	0.47 ± 0.03	0.36 ± 0.02	0.32 ± 0.02	0.42 ± 0.02	0.29 ± 0.01	0.32 ± 0.03
C16:2 n-4	0.18 ± 0.02	0.34 ± 0.02	0.11 ± 0.02	0.13 ± 0.03	0.17 ± 0.02	0.22 ± 0.03	0.18 ± 0.02	0.25 ± 0.02
C16:3 n-3	0.10 ± 0.01	0.12 ± 0.01	0.10 ± 0.01	0.07 ± 0.01	0.05 ± 0.01	0.07 ± 0.01	0.06 ± 0.01	0.06 ± 0.01
C18:2 n-6	16.60 ± 0.86	16.85 ± 0.66	16.90 ± 0.67	16.91 ± 0.88	17.98 ± 0.87	19.22 ± 0.86	17.54 ± 0.94	16.93 ± 0.88
C18:3 n-3	55.60 ± 0.77	49.59 ± 1.12	52.75 ± 0.74	53.24 ± 1.29	49.41 ± 1.33	46.23 ± 1.20	51.75 ± 1.36	45.65 ± 1.21
Sum of PUFA	72.88 ± 0.36	67.51 ± 0.83	70.32 ± 0.64	70.71 ± 0.77	67.93 ± 0.67	66.17 ± 0.63	69.81 ± 0.57	63.21 ± 1.40
Leaf Total Fatty acids	2.16 ± 0.07	1.22 ± 0.12	1.53 ± 0.05	2.01 ± 0.13	1.16 ± 0.04	1.19 ± 0.04	1.30 ± 0.11	1.22 ± 0.06

The average of daily SST data through the 10 days before sampling is reported for each site.

**Figure 2:**
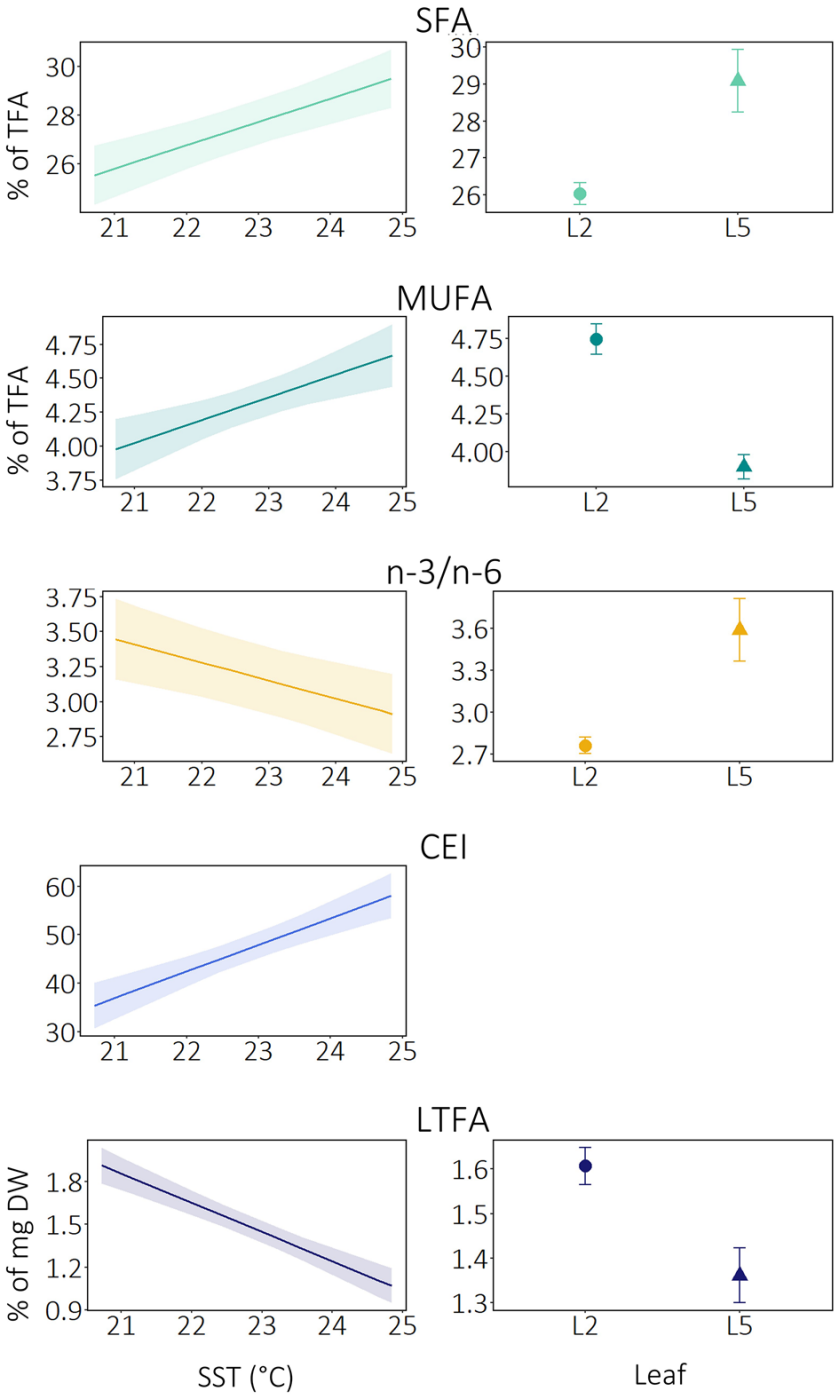
Generalized least squares regressions of SFA, MUFA, n-3/n-6, CEI and LTFA with SST (Sea Surface mean Temperature) and Leaf (L2 vs L5).

As expected, FA groups were all related to the SST (Table 2 and Fig. 2). However, the best relationships described by GLS models also incorporated the leaf age within the shoot (but not their interaction, SST × Leaf) for all response variables, except CEI (Table 2 and Fig. 2). Moreover, the AIC indicated that the variance structure for leaf improved the models, compared to the linear regression model.

**Table 2:** Results of the GLSs on the FA groups (SFA, MUFA, n-3/n-6, CEI and LTFA,): SST = sea surface mean temperature (10 days before sampling) and Leaf (L2 vs L5).

	Value	St. error	t-value	*P* value
SFA				
Intercept	4.200	4.474	0.939	0.3503
SST	0.957	0.196	4.890	**< 0.0001**
Leaf	3.056	0.896	0.896	**0.001**
MUFA				
Intercept	0.938	1.034	0.907	0.3669
SST	0.167	0.045	3.706	**0.0004**
Leaf	− 0.845	0.128	− 6.589	**< 0.0001**
n-3/n-6				
Intercept	5.718	0.933	6.127	**< 0.0001**
SST	− 0.130	0.041	− 3.175	**< 0.0001**
Leaf	0.827	0.232	3.569	**< 0.0001**
CEI				
Intercept	− 78.546	21.500	− 3.653	**< 0.0001**
SST	5.500	0.940	5.850	**< 0.0001**
LTFA				
Intercept	6.688	0.521	12.840	**< 0.0001**
SST	− 0.224	0.023	− 9.803	**< 0.0001**
Leaf	− 0.222	0.074	− 3.018	**0.0033**

In bold the significant values.

Particularly, increases in SST corresponded to an increase of the predicted values of SFA, MUFA, and CEI, while the opposite trend was found for n-3/n-6 and LTFA (Table 2 and Fig. 2). PUFA and PUFA/SFA were negatively associated to SST, due to their negative correlation with SFA. Similarly, n-3 and n-6 were associated to SST, the former negatively and the latter positively, due to the correlation with n-3/n-6 (Table 2 and Fig. 2). Significant variations were also found between the two leaves (L2 and L5): higher values of MUFA (*P* < 0.0001) and LTFA (and thus of PUFA, PUFA/SFA, n-3 and n-6; *P* = 0.0033) were associated to L2, while higher values of SFA and n-3/n-6 were associated to L5 (*P* = 0.001 and *P* < 0.0001, respectively; Fig. 2).

In addition, L2 FA profiles not only depended significantly on the site (which corresponded to differences in SST), but also on the area within each site; the exceptions were LTFA (*P* < 0.0001) and n-3/n-6 (*P* = 0.00018) which differed only among sites and areas, respectively (Table 3 and Fig. S2).

**Table 3:** Results of the ANOVAs on the effects of Site and Area on the L2 (second leaf) FA groups (SFA, MUFA, n-3/n-6, CEI and LTFA).

	df	F value	*P* value
*SFA*			
Site	7	10.02	**< 0.0001**
Area (S)	16	2.96	**0.00189**
Residuals	48		
MUFA^T^			
Site	7	5.03	**0.00358**
Area (S)	16	2.07	**0.02675**
Residuals	48		
n-3/n-6			
Site	7	1.30	0.30965
Area (S)	16	3.77	**0.00018**
Residuals	48		
CEI^T^			
Site	7	3.57	**0.01655**
Area (S)	16	3.52	**0.00037**
Residuals	48		
LTFA^T^			
Site	7	26.57	**< 0.0001**
Area (S)	16	1.62	0.09931
Residuals	48		

In bold the significant *P* values, while T indicates log(x) transformed data.

## Discussion

This study was driven by the need to estimate if the variability of seagrass leaf FA profiles due to the thermal gradient could be also influenced by leaf material and spatial variability, to identify seagrass trait responses and good sampling practices to be used as warming descriptors. Overall, all the hypotheses tested were confirmed.

**Figure 3:**
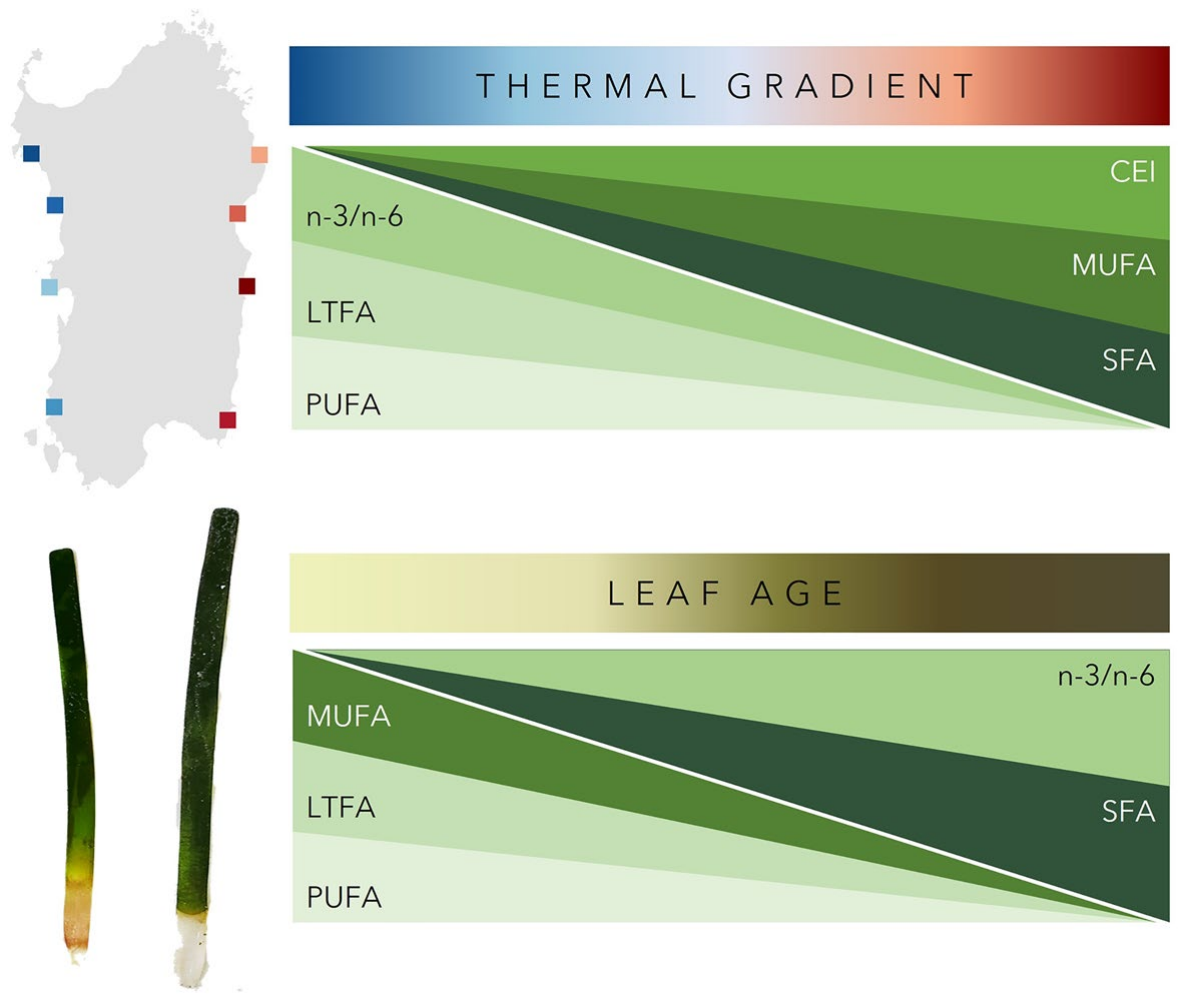
Summary of the results. Fatty acid profiles on the second and fifth leaf of the shoots were measured along a natural sea surface temperature gradient. A reduction in leaf total fatty acid content (LTFA), polyunsaturated fatty acids (PUFA), omega-3/omega-6 PUFA (n-3/n-6) and an increase in saturated fatty acids (SFA), monounsaturated fatty acids (MUFA) and carbon elongation index (CEI, C18:2 n-6/C16:2 n-6) were associated to higher temperatures. A reduction in MUFA, LTFA and PUFA and an increase in n-3/n-6 and SFA were associated to older leaves.

A clear association of the FA composition of *P. oceanica* early summer plants with temperature gradient was highlighted. The spatial association between FA composition in *P. oceanica* and sea water temperature suggests that future warming is predicted to influence FA remodelling, reflecting the expectations based on information gained for this species and other seagrasses^34,35,46,62^. A first common pattern observed in primary producers is the overall reduction of LTFA content with temperature increase, suggesting a possible arrangement of energetically rich substrates as a compensation measure due to the environmental stress^35,63^. Specifically, for both second and fifth leaves, temperature gradient also impacted the FA groups composition (Fig. 3).

Differences in FA profiles in plant cells membrane lipids could be interpreted as a required metabolic pathway to retain the photosynthetic thermostability and acclimation to temperature conditions^38^. Therefore, the overall reported increase in SFA and MUFA and the following decrease in PUFA and PUFA/SFA (heat stress index) with warmer temperatures can be ascribable to different homeoviscous conditions as an effort to preserve membrane fluidity properties and electron transport^40,63–65^. Higher proportions of SFA would be produced to provide optimal cell membrane structural rigidity, counteracting the increasing fluidity produced in warmer conditions^35,66^. Consequently, in natural environment warm-acclimated populations exhibit higher levels of SFA than their cool-acclimated counterparts. Similarly, the decrease in polyunsaturated n-3 group (C16:3 n-3 and C18:3 n-3) relative to n-6 group (C16:2 n-6 and C18:2 n-6), and thus the n-3/n-6 (seagrass photosynthetic productivity index), could reflect the increase in SFA and MUFA (which are substrates of polyunsaturated FA) to maintain membrane fluidity, adjusting their metabolism in direct response to the warming stress, as mentioned above.

Furthermore, we evidenced a significant source of intra-shoot variability in FA composition between the second and fifth leaf of the same shoot. Specifically, the contribution of different FA groups (except for the CEI) changed and leaf 5 contained higher SFA and n-3/n-6 and lower PUFA, MUFA and LTFA content than leaf 2. As higher values of PUFAs are associated to higher photosynthetic activity^46,64^, this result could suggest that PUFAs are proxy of productivity of seagrass plants, especially in younger leaves where photosynthetic activity is higher^53,57^. Therefore, depending on the influence of temperature, leaf-type effect on specific functioning and metabolism would be missed if the seagrass material collected is not accurately decided by a procedural protocol and each leaf within a shoot was treated as a possible replicate^55^. Indeed, the amount of variability in FA groups associated to the range of temperature considered is very similar to that occurring between the leaves of the shoot, supporting the need of carefully evaluating the plant material to collect for the analysis not to add undesired variability to the results. Thermo-tolerance of plants is known to be strongly influenced by leaf age and life span with a general higher resistance of the more mature leaves to the heat^67–69^. In monocotyledonous plants a strong vertical age gradient is also present within the same leaf, as the undifferentiated cells are located at the base of the blade and the most mature cells occurring at the tips^70^. The gradient of leaf developmental stages reflects the differences in biochemical and physiological processes along the leaf length and across leaves^55,69,71^. The higher levels of FAs associated to photosynthesis in the second leaf (younger or intermediate^57^) suggest this plant material is more suitable to detect temperature influence on seagrass species. The variation in photosynthetic activity is considered one of the most sensitive seagrass physiological responses to heat^69^, and this study supports the hypothesis that the effect of temperature on the FA profiles is consistent between leaves. These findings are in accordance with previous studies that investigated the nutritional role of different leaves in the same shoot^57^. Overall, this evidence fosters the need of accurately selecting the seagrass material to produce comparative evaluations and possibly rely on solid descriptors of heat stress.

The much less obvious result is the fact that we have identified associations of FA groups with SST in a very field range of mean temperature (about 4 °C) and a relatively restricted spatial range. Descriptive studies that investigated the remodelling of FA composition in seagrasses have generally dealt with wider temperature ranges, often larger than 10 °C, both because of either wide temporal or latitudinal ranges (e.g.^39,43^). Some manipulative experiments have instead tested the effect of a 4 °C temperature increase (treatment similar to the range considered in this study and comparable to climate change scenarios) in a relatively short time^34,35,43^ to simulate a warming event. Although different in the time of warming, the response of FA in plants subjected to the temperature treatment in mesocosms resembled the FA composition associated to SST found in this study in untouched plants.

Overall, obtaining biochemical changes in *P. oceanica* plants living at sites located within a gradient of thermal environment was possible underlying the high sensitivity of FA groups to temperature^62^. This issue is also corroborated by the fact that the association of FA with temperature and leaf age were identified despite of the significant spatial variability at scale of 100 s of m, as changes in all FA groups (except for LTFA) were found among areas at the same temperature. This result confirms how environment-induced variation can affect FA profiles, as Schmid et al.^72^ described for seaweeds. If disregarded, the role of temperature may be confounded by effects due to local conditions; therefore, the choice of dealing with a hierarchical sampling design (several seagrass individuals and environmental factors within replicated areas) should be encouraged.

This study proposed the response of several *P. oceanica* FA groups as possible descriptors in a thermal summer gradient resembling future warming scenario. Although we are aware that the use of satellite-derived SST data as proxy of thermal environment at 10 m depth is less accurate than in-situ measures, we highlight the reliability of this approach when testing wide range gradients as in this study, where the effect of variations in summer Mediterranean temperature, resembling the future climate changes, on *P. oceanica* biochemical response in the field was evaluated. Nevertheless, further research is needed to predict global warming effects on plant species and define if FA profiles are good candidate to explain species response to the heat stress, whose adjustment to temperature shifts needs to be deeply investigated. However, the significant role of spatial variability and plant material on the highly sensitive response of FA profiles of *P. oceanica* leaves here evidenced, suggest the necessity that methodology and sampling design in seagrass studies and in monitoring plans will be carefully decided to obtain reliable indicators.

## Methods

### Study location

**Figure 4:**
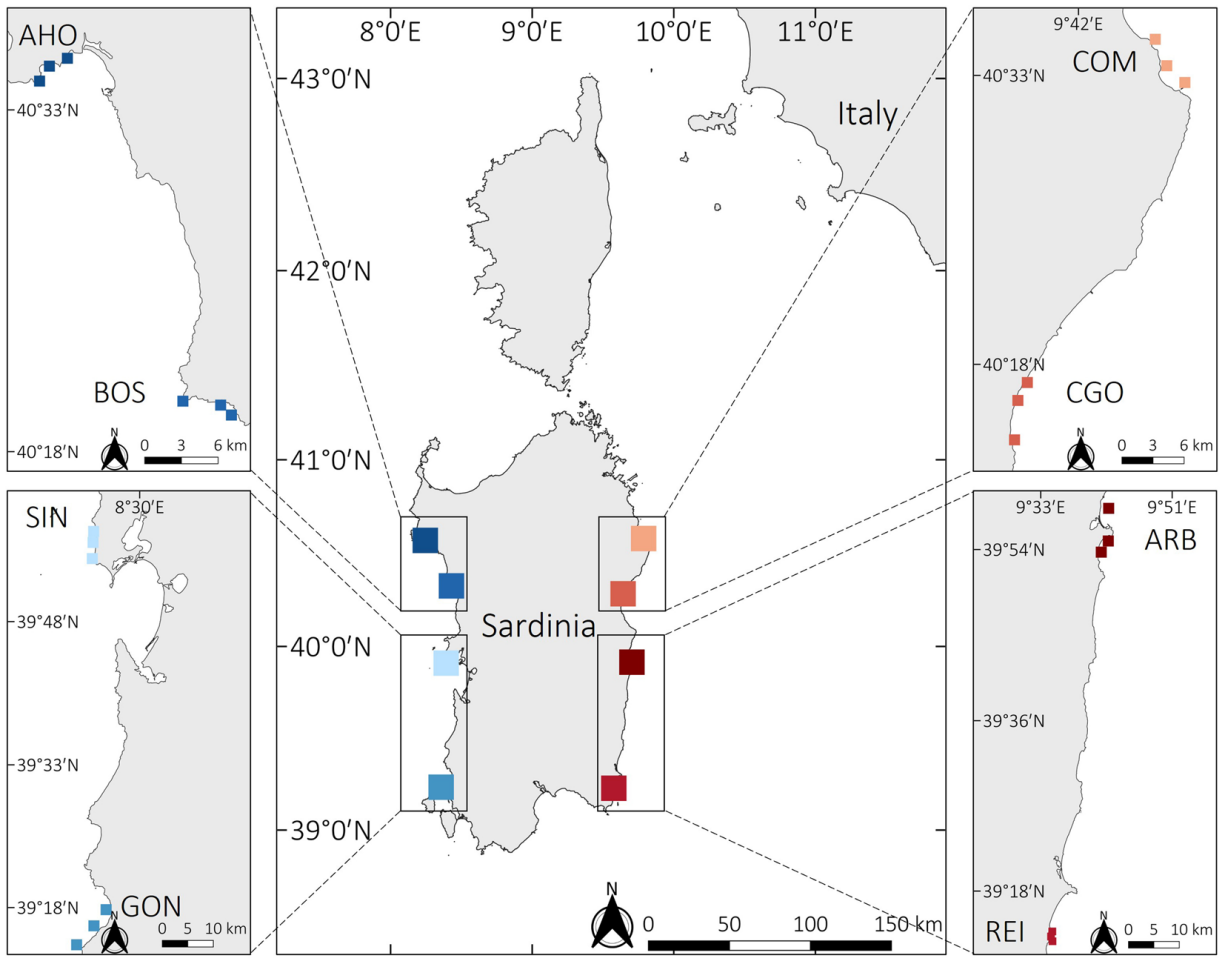
Sites of the study along the Sardinian Coasts. AHO = Alghero, BOS = Bosa, SIN = Penisola del Sinis, GON = Gonnesa, COM = Capo Comino, CGO = Cala Gonone, ARB = Arbatax, REI = Costa Rei. Zoomed maps show the three study areas within each site. Map produced with QGIS 3.16 software (https://www.qgis.org/de/site/).

This study was done in Sardinia (Italy, western Mediterranean Sea, Fig. 4) where differences in water temperature between the western and eastern coasts are evident in a very narrow range of latitude. In fact, the western coastline is directly affected by Atlantic waters through the Western Mid-Mediterranean Current and is also influenced by coastal upwellings^73^, while the eastern coast is affected by the warm Algerian Current^74^.

*P. oceanica* meadows were sampled in four sites of the west (Alghero = AHO, Bosa = BOS, Penisola del Sinis = SIN, and Gonnesa = GON) and four of the east coast (Capo Comino = COM, Cala Gonone = CGO, Arbatax = ARB, and Costa Rei = REI), from 40°34’ to 39°15’N. The seagrass meadows sampled were all dense (on average 581 shoots/m^2^^25^), and far from anthropogenic disturbances. Collection of samples followed a hierarchical sampling design, as at each site three areas 100 s of m apart were randomly selected (Fig. 4).

### Seagrass sampling

**Figure 5:**
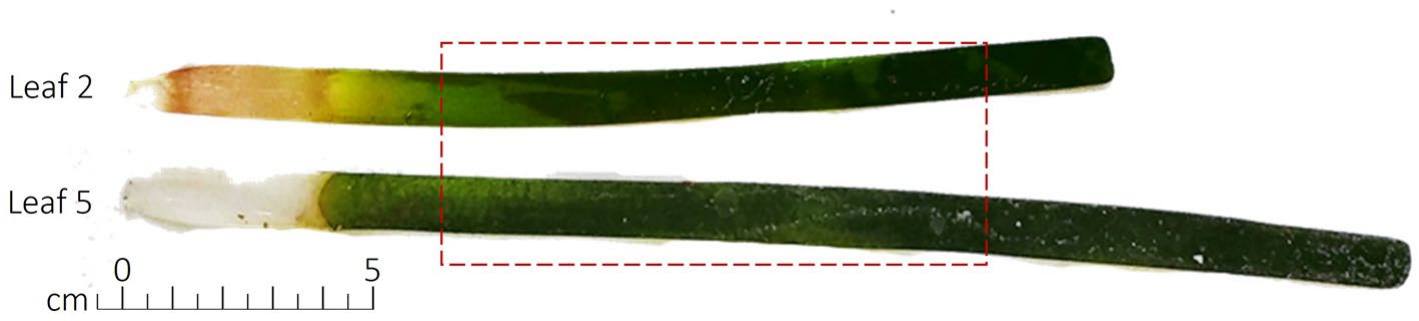
*Posidonia oceanica* leaf portion selected for the fatty acid analysis on leaf 2 and 5.

Samplings were done in meadows at 10 m of depth, from the 20^th^ of June to the 10^th^ of July 2020. At each area within the sites three *P. oceanica* orthotropic shoots were sampled to minimise the impact on the threatened species and the 72 shoots collected were transported to the laboratory and stored frozen at − 20 °C. The authors declare that shoot sampling was non-lethal for the seagrass meadow, following the guidelines approved by the Marine Strategy Framework Directive^75^ for the monitoring program issued by the Italian Institute for Environmental Protection and Research (ISPRA, https://www.isprambiente.gov.it/files/icram/scheda-metodologia-posidonia-new.pdf). *Posidonia oceanica* (L.) Delile shoots were identified by Arianna Pansini and deposited as voucher specimens at the University of Sassari Herbarium (SS, collection 2000/, ID number: SS#14159-SS#14166). In the laboratory, the intermediate second leaf, more than 5 cm long without basal sheath (L2 from 72 shoots) and the adult fifth leaf, more than 5 cm long with basal sheath (L5, from 24 shoots, one per area) of each shoot, were identified washed in filtered sea water to remove extraneous materials, cleaned from epiphytes, and kept frozen at − 20 °C. For each leaf, the photosynthetically active part (the middle section, from ~ 7 cm of distance from the bottom to 20 cm of length, Fig. 5) was selected and dried for 72 h at ambient temperature and once completely dried samples were kept frozen in zip lock bags containing silica gel^39^. Before analyses, the leaves were first freeze-dried using a Labconco Freezone 6 freeze dryer machine (USA) and finely ground into powder using a homogenizer (Beadmill 4 machine at 5 m s^−1^ for 3 min).

### Fatty acid extraction

Fatty acids methyl esters (FAMEs) were determined by direct transmethylation of ~ 10 mg of powdered biomass with dry methanol containing 2% (v/v) H_2_SO_4_. To quantify total and individual FA composition and contents, the standard Nonadecanoic acid, C19:0 (20 μl, 0.8 mg m^−1^, ≥ 98, catalogue no. N5252, Sigma Aldrich, St Louis, USA) was added as an internal standard before the transmethylation process. To prevent oxidation, vials were sealed with nitrogen gas before being heated at 80 °C for 2 h under stirring conditions. After transmethylation, 1 mL of Milli-Q water was added, and FAMEs were extracted using 0.25 mL of n-hexane. Identification of FAMEs was conducted using a Clarus 500 Gas Chromatograph (Perkin Elmer Instruments, USA) equipped with a flame ionization detector and a fused silica capillary column (SP-2330, 0.25 mm × 30 m × 0.2 μm, Supelco, catalogue 24019). Identification of FAME was achieved by co-chromatography with commercially available FAME certified standard material (Supelco 37 Component FAME Mix, catalogue CRM47885).

The FA composition was determined and the proportion was expressed as relative contribution (%) of the grouped fatty acid SFA (sum of C14:0, C15:0, C16:0, C18:0, C20:0), MUFA (sum of C16:1 n-9, C16:1 n-7, C18:1 n-9, C18:1 n-7), and PUFA (sum of C16:2 n-6, C16:2 n-4, C16:3 n-3, C18:2 n-6, C18:3 n-3) groups. Moreover, the following FA ratios were considered: PUFA/SFA as an index of thermal stress^35,39^, n-3/n-6 (the sum n-3 PUFA/sum of n-6 PUFA as an index of seagrass photosynthetic productivity^39^), and an additional biomarker proposed as carbon elongation index (CEI, C18:2 n-6/C16:2 n-6). Finally, leaf total FA content (LTFA) was calculated and expressed in % mg DW.

### Temperature data

For each site the SST for the ten days preceding the specific *P. oceanica* shoot collection day (the average response time of FA profiles to environmental factors^38,43,44,63^), was obtained by the Group for High Resolution Sea Surface Temperature (GHRSST) daily (1 km resolution, G1SST dataset produced by JPL NASA, https://coastwatch.pfeg.noaa.gov/erddap/griddap/jplMURSST41.html). SST was used as proxy of the site 10 m subtidal temperature^21,25,26,44,76,77^ and due to the resolution, it allowed assuming the same thermal conditions for the three areas within each site. For each site, daily SST data were averaged through the ten days before sampling. Despite satellite-derived SST data are less appropriate than in-situ measures, they allow investigating the interaction between biotic and abiotic variables on ecological dynamics^78^.

### Data analysis

The associations between FA composition and content (response variables) and SST (continuous variable), leaf (factor with two levels: L2 and L5), and their interaction were tested using Generalized Least Square (GLS) models. GLSs are weighted linear regressions which consider the inequality of variance in the observations, allowing for heterogeneity. Before running the models, the collinearity between the response variables was inspected with pair-plots, and variance inflation factors (VIFs) were calculated. Analyses were only run on SFA, MUFA, n-3/n-6, CEI and LTFA; in fact, PUFA and PUFA/SFA were negatively correlated with SFA, as well as n-3 and n-6 with n-3/n-6 (positively and negatively, respectively). Analyses were performed in R^79^ using the nlme package^80^. Because FA data presented variance heterogeneity among the factor, the variance leaf structure with different spread per level of the factor (“VarIdent” Variance Structure) was included into the GLS analyses^81^. The best model was selected according to minimum Akaike information criterion (AIC, Supplementary Table S1). Model validations were run calculating and plotting the normalized residuals against the (i) fitted values, (ii) each explanatory variable in the model, and (iii) each explanatory variable not in the model (Supplementary Fig. S1)^81^.

To estimate relevant scales of spatial variability in FA composition, for each response variable (SFA, MUFA, n-3/n-6, CEI and LTFA) obtained only from L2, a two-way ANOVA was run to test the effect of site (AHO, BOS, GON, SIN, COM, CGO, REI, ARB) and area (three levels) random and nested in site. Homogeneity of variances was checked by Cochran’s test.

## Research involving plants

The authors declare that the seagrass samplings have been carried out in accordance with relevant guidelines and regulations.

## Supplementary Information

Supplementary Information.

## Data Availability

The datasets generated and analysed during the current study are available from the corresponding author on reasonable request.
