# Supplementary material for: Sources of variability in seagrass fatty acid profiles and the need of identifying reliable warming descriptors
Source: Sci Rep. 2023 Jun 20;13:10000. doi: 10.1038/s41598-023-36498-2 (PMC10282067; doi:10.1038/s41598-023-36498-2)

Identifying sources of variability in seagrass fatty acid profiles to rely on valuable warming descriptors

Arianna Pansini, Pedro Beca-Carretero, Maria J. González, Gabriella La Manna, Isabel Medina, Giulia Ceccherelli

Table S1. Akaike's Information Criterion (AIC) model selection of GLS for *Posidonia oceanica* Fatty acid response variables (SFA, MUFA, n-3/n-6, CEI and LTFA) to sea surface temperature (SST), and Leaf (L2 and L5). Δ AIC is the difference in AIC score between the best model and the model being compared.

	df	AIC	Δ AIC
SFA			
SST × Leaf + change in variance with Leaf	6	482.2295	0
SST × Leaf + change in variance with SST	5	489.6576	7.4281
SST × Leaf	5	490.5321	8.3026
MUFA			
SST × Leaf + change in variance with Leaf	6	216.262	0
SST × Leaf + change in variance with SST	5	227.669	11.407
SST × Leaf	5	228.6359	12.3739
n-3/n-6			
SST × Leaf + change in variance with Leaf	6	200.4902	0
SST × Leaf	5	222.135	21.6448
SST × Leaf + change in variance with SST	5	222.1635	21.6733
CEI			
SST × Leaf + change in variance with Leaf	6	747.1552	0
SST × Leaf + change in variance with SST	5	748.8411	1.6859
SST × Leaf	5	750.1178	2.9626
LTFA			
SST × Leaf	5	70.74717	0
SST × Leaf + change in variance with SST	5	72.1382	1.39103
SST × Leaf + change in variance with Leaf	6	72.64259	1.89542

Fig. S1 Model validation plots of the residuals against fitted values, sea surface temperature (SST), and Leaf (L2 and L5) and *Posidonia oceanica* Fatty acid response variables (SFA, MUFA, n-3/n-6, CEI and LTFA).

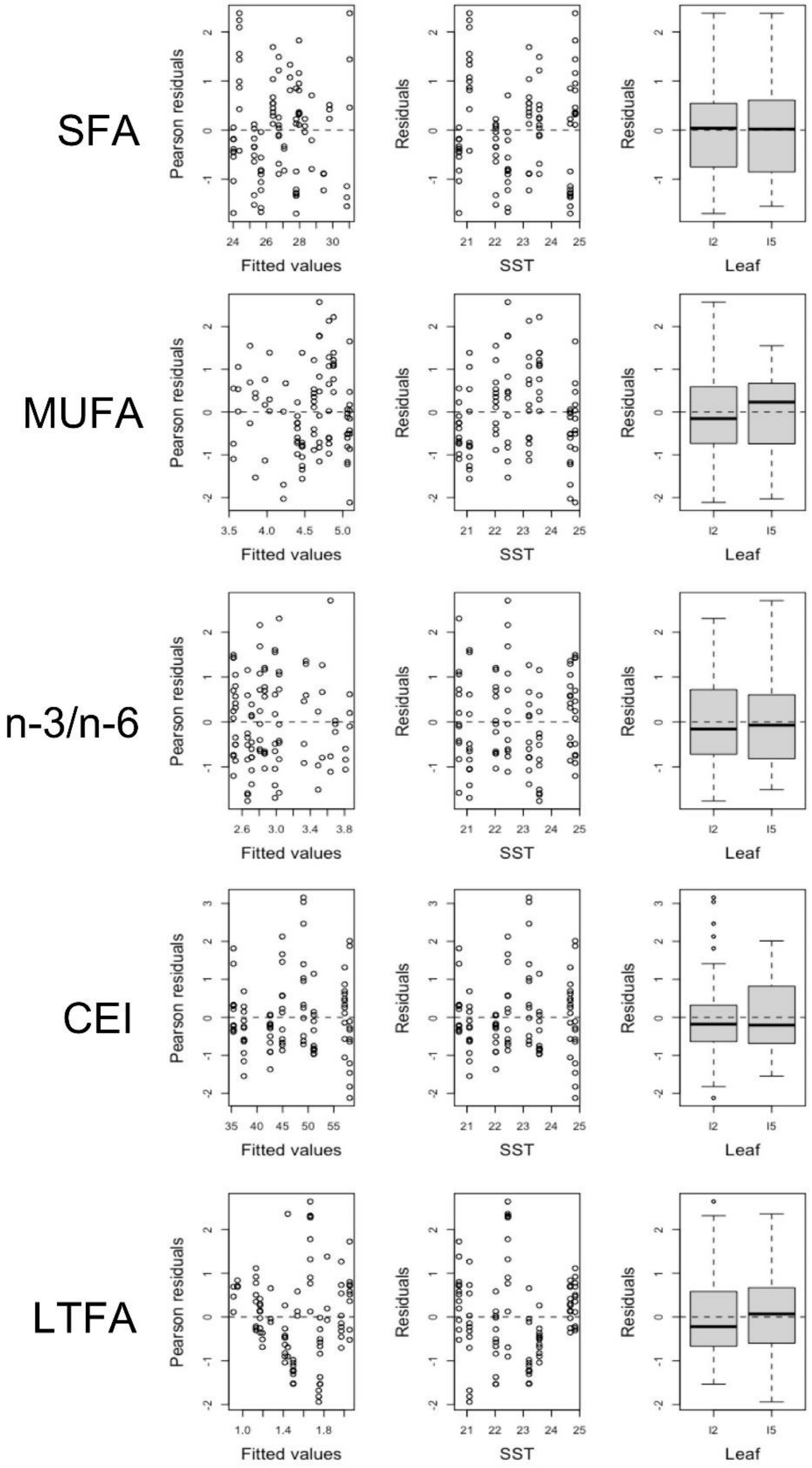

Fig. S2 Box-plots of *Posidonia oceanica* Fatty acid response variables (SFA, MUFA, n-3/n-6, CEI and LTFA). For each location data of the three areas are shown (n=3)

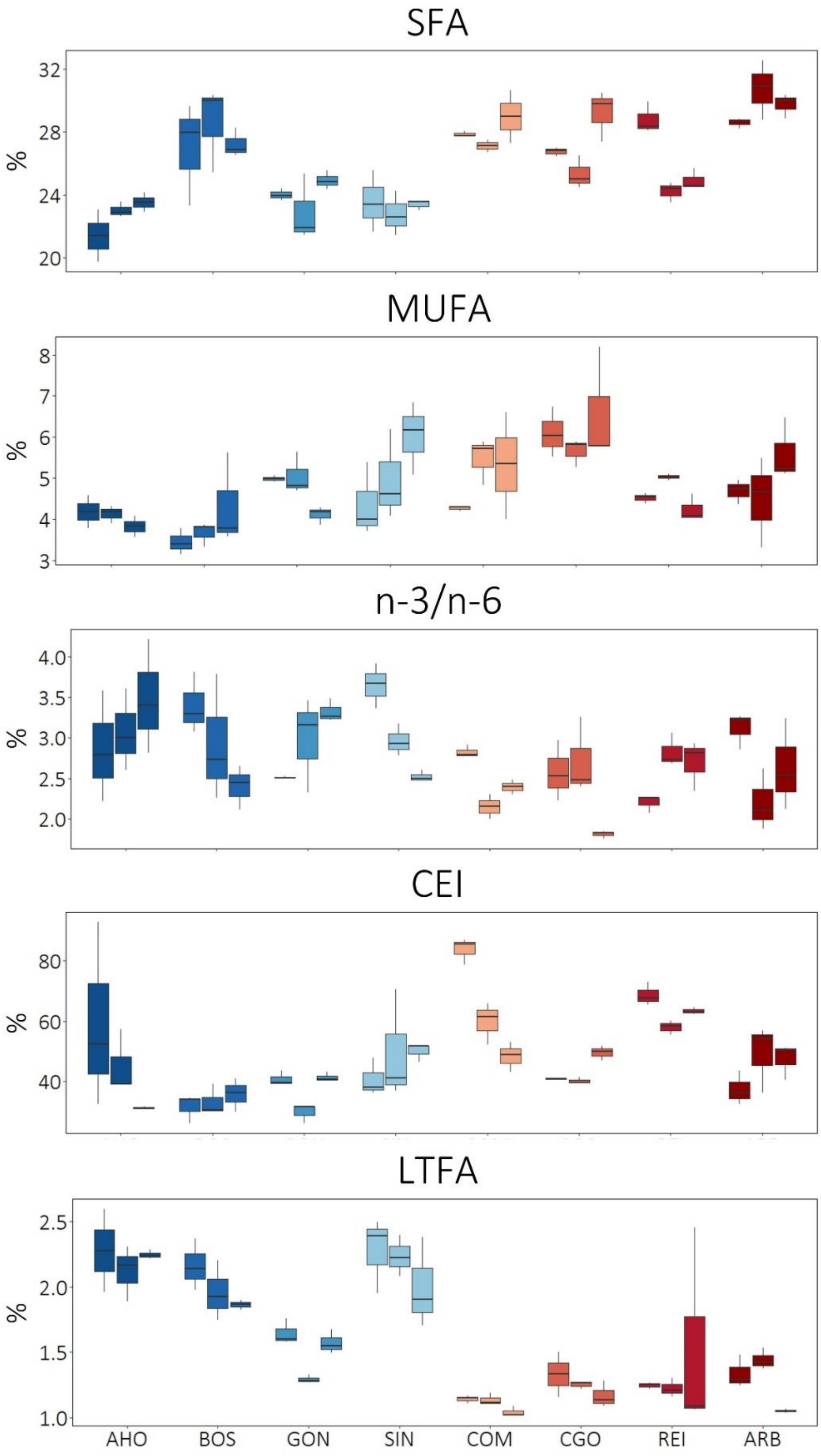

Supplement: Supplementary file 1 — Supplementary Information. [file 41598_2023_36498_MOESM1_ESM.pdf]
